# Surface Sampling Collection and Culture Methods for *Escherichia coli* in Household Environments with High Fecal Contamination

**DOI:** 10.3390/ijerph14080947

**Published:** 2017-08-22

**Authors:** Natalie G. Exum, Margaret N. Kosek, Meghan F. Davis, Kellogg J. Schwab

**Affiliations:** 1 Department of Environmental Health and Engineering, Bloomberg School of Public Health, Johns Hopkins University, Baltimore, MD 21205-2179, USA; nexum1@jhu.edu (N.G.E.); mdavis65@jhu.edu (M.F.D.); 2 Department of International Health, Johns Hopkins Bloomberg School of Public Health, Baltimore, MD 21205-2179, USA; mkosek@jhmi.edu; 3 Asociación Benéfica Proyectos de Informática, Salud, Medicina, y Agricultura (A.B. PRISMA), Iquitos, Peru

**Keywords:** fomites, surfaces, sanitation, fecal contamination, environmental sampling, *Escherichia coli*, infections/transmission, methods/microbiology

## Abstract

Empiric quantification of environmental fecal contamination is an important step toward understanding the impact that water, sanitation, and hygiene interventions have on reducing enteric infections. There is a need to standardize the methods used for surface sampling in field studies that examine fecal contamination in low-income settings. The dry cloth method presented in this manuscript improves upon the more commonly used swabbing technique that has been shown in the literature to have a low sampling efficiency. The recovery efficiency of a dry electrostatic cloth sampling method was evaluated using *Escherichia coli* and then applied to household surfaces in Iquitos, Peru, where there is high fecal contamination and enteric infection. Side-by-side measurements were taken from various floor locations within a household at the same time over a three-month period to compare for consistency of quantification of *E. coli* bacteria. The dry cloth sampling method in the laboratory setting showed 105% (95% Confidence Interval: 98%, 113%) *E. coli* recovery efficiency off of the cloths. The field application demonstrated strong agreement of side-by-side results (Pearson correlation coefficient for dirt surfaces was 0.83 (*p* < 0.0001) and 0.91 (*p* < 0.0001) for cement surfaces) and moderate agreement for results between entrance and kitchen samples (Pearson (0.53, *p* < 0.0001) and weighted Kappa statistic (0.54, *p* < 0.0001)). Our findings suggest that this method can be utilized in households with high bacterial loads using either continuous (quantitative) or categorical (semi-quantitative) data. The standardization of this low-cost, dry electrostatic cloth sampling method can be used to measure differences between households in intervention and non-intervention arms of randomized trials.

## 1. Introduction

According to the Joint Monitoring Programme for Water Supply and Sanitation 2017 Update, there is now an estimated 4.5 billion people that lack access to safely managed sanitation [1]. In communities where fecal waste is unsafely managed and enters the environment untreated, high concentrations of pathogens are easily transmitted through multiple environmental reservoirs [2]. Surfaces are one of these reservoirs that may significantly contribute to the transmission of pathogens. Both human and animal sources of fecal contamination have been found to exist on surfaces (including floors) in the household environment of low-income communities [3], contributing to increases in disease risk to humans [4]. To assess the risk that this environmental reservoir presents, accurate measurements of the pathogen loads are necessary for exposure assessment; however, the traditional surface sampling swabbing techniques have been shown to have an overall poor performance [5].

*Escherichia coli* is a standard indicator bacterium to assess environmental fecal contamination from both human and animal sources, and has been used to evaluate household floors and surfaces in low-income settings [6,7,8,9]. Most methods for the surface sampling of *E. coli* involve a swab and wetting solution. While variations on this method may increase relative test sensitivity, overall bacterial recovery with the wet swab method has a low efficiency for epidemiologic analysis [5]. Moore et al. [5] determined that the optimum sampling efficiency (including removal from the surface and recovery efficiency from the swab) that can be achieved by combining various swab wetting solutions and their components was no more than 34%. Therefore, there is a need to improve this low efficiency and develop an optimized and validated protocol. An important first step is establishing a sampling method that can be consistently used across studies evaluating the impact of interventions to reduce fecal contamination in low-income communities.

This paper presents a method for the measurement of *E. coli* on surfaces that can be applied across a variety of surface types to improve the quantification of fecal pathogens. We evaluate the effectiveness of a dry electrostatic cloth (Swiffer™; Proctor & Gamble, Cincinnati, OH, USA) sampling method in peri-urban Peruvian household environments with high fecal contamination. This strategy was adapted from one developed for sampling home environmental contamination with *Staphylococcus aureus* from dry dust wipe samples [10]. We provide laboratory quantification of the recovery efficiency from the cloths and apply the technique to sampling floors in the homes of children enrolled in the Etiology, Risk Factors, and Interactions of Enteric Infections and Malnutrition and the Consequences for Child Health and Development (MAL-ED) cohort study in Iquitos, Peru [7,11].

## 2. Materials and Methods

### 2.1. Laboratory Experiment: E. coli Inoculum Preparation

For laboratory phase experiments, stocks of Nalidixic acid-resistant *E. coli* CN-13 (ATCC 700609) were generated by inoculating tryptic soy broth (TSB) (Invitrogen, Carlsbad, CA, USA) containing 1% Nalidixic acid solution (Sigma, St. Louis, MO, USA) with one loop (5 μL) of frozen stock followed by incubation on a rotary shaker at 37 °C with mixing at 115 rpm for 16–20 h to produce a stationary-phase stock. *E. coli* CN-13 stocks were enumerated by plating 100 μL, 10-fold serial dilutions in phosphate-buffered saline (PBS) on 1.5% tryptic soy agar Petri dishes with 1% Nalidixic acid, spreading the samples evenly over the plate with glass beads, incubating inverted overnight at 37 °C, with subsequent counting of bacterial colonies.

### 2.2. Laboratory Experiment: Recovery of E. coli from Directly Inoculated Cloths

To estimate recovery efficiency from cloth samples, 538 cm^2^ cloths were first cut into quarter sizes (134 cm^2^), wrapped in autoclave paper, and sterilized by autoclaving. Cloths were then spiked with 100 μL of PBS containing approximately 100 *E. coli* CN-13 bacteria. After ensuring that the inoculum was absorbed into the cloth, it was placed in a 700 mL Whirl-Pak bag (Nasco, Fork Atkinson, WI, USA), and eluted as previously descried in Exum et al. [7]. Briefly, 100 mL of sterile 0.1% Peptone buffer was added into the Whirl-Pak bag and the cloth was vigorously shaken inside the bag for one minute to release the *E. coli* cells into the buffer solution. The cloth was then aseptically wrung out inside the Whirl-Pak bag by pinching the cloth between the interior sides of the bag to release as much buffer solution as possible, after which the cloth was then removed. A positive control for each experiment was prepared by spiking 100 μL of PBS solution containing approximately 100 *E. coli* CN-13 bacteria directly into 100 mL of sterile 0.1% Peptone buffer, bypassing the step of inoculating the dry cloth. For both Whirl-Pak bags containing buffer solution with *E. coli* from inoculated cloths and the positive controls, all 100 mL of these samples were filtered through 47-mm diameter, 0.45-μm pore-sized filter membranes (EMD Millipore, Billerica, MA, USA).

*E. coli* in the buffer for the experiments and controls were enumerated with membrane filtration following United States Environmental Protection Agency Method 1604 [12] using m-coliblue24 commercial media (HACH, Loveland, CO, USA). Percent recovery for each experiment was calculated by dividing the Colony Forming Units (CFU) recovered from the cloths by the CFU recovered from the positive control.

### 2.3. Field Application: Household Sampling

In May and June 2015, surfaces in households with children enrolled in the MAL-ED study [11] at the Iquitos, Peru (3°47′ S, 73°20′ W) site were sampled using autoclaved dry electrostatic cloths. The same households were revisited in August and September 2015 for a second phase of sampling to evaluate heterogeneity across time within household locations. To evaluate heterogeneity across different locations within the household, two 900 cm^2^ floor locations were sampled at the front and back of the housing structures: the entrance floor and kitchen floor.

To quantify the heterogeneity of *E. coli* on a floor area, a subset of homes visited had duplicate samples taken side-by-side in the entrance area. Floor surface type (e.g., dirt, cement) was determined by inspection. At each sampling site, a collector wearing new nitrile gloves aseptically obtained an autoclaved cloth and the dry cloths were passed over a 30 cm by 30 cm area (900 cm^2^). The cloth was then placed in a sterile Whirl-Pak bag containing 5 mL of Milli-Q ultra-pure water to reduce potential bacterial die-off and transported to the lab at 4 °C. The 5 mL of Milli-Q ultra-pure water was added to the Whirl-Pak bag from an individually packaged Falcon tube and disposed of after use to ensure sterility. All samples were processed within 6 hours of collection. Cloths were processed as outlined above. High bacterial loads were anticipated but because the concentration of bacteria was unknown, undiluted, 10-fold, 100-fold, and 1000-fold dilutions from each sample were filtered in 100 mL of sterile 0.1% Peptone buffer, and recovered *E. coli* on the filter membrane were cultured as previously described for the laboratory experiments. Ethical approval for the household sampling was given by the Johns Hopkins Institutional Review Board as well as from the partner organization, PRISMA Benefit Association, in Iquitos, Peru.

### 2.4. Field Application: Data Analysis

*E. coli* CFU enumerated from each cloth were log_10_-transformed to meet the normality assumptions for the Pearson correlation coefficient. Log_10_-transformed *E. coli* concentrations were classified into quartiles and analyzed via the Kappa statistic to evaluate whether interpretation was similar for both quantitative and semi-quantitative analysis [13]. Data are reported as log_10_ *E. coli* CFU/900 cm^2^. Results were compared between duplicate entrance floor samples within the same household, between entrance and kitchen floor samples within the same household at the same time, and between the first and second sampling at the same location at the same household taken three months later using Pearson correlation coefficients. Data were also stratified by floor type to estimate the effects from various surfaces on bacterial survival on floors. Data were categorized into quartiles to be interpretable with Kappa statistics and Kappa interpretations, which were calculated for the categorical data with squared weights according to criteria by Landis and Koch [13]. The data were processed and analyzed using R software version 3.0.3 (R-FSC, Vienna, Austria) with the Kappa statistic analyzed using the “irr” package [14] and the “cor.test” function for correlation.

## 3. Results

### 3.1. Laboratory Quantification of E. coli Recovery

For the laboratory phase of the methods development, *E. coli* recovery values were based on 39 replicates. The average number of *E. coli* bacteria colonies eluted off of the cloths was 107 CFU (95% Confidence Interval (CI): 74, 155). The average recovery of *E. coli* colonies from the cloths was 105% (95% CI: 98%, 113%).

### 3.2. Field Application to Evaluate Heterogeneity within Household Locations

Table 1 provides the spatial agreement of results for recovery of *E. coli* from household surfaces using the field application of the dry cloth sampling method. The surface types for the floors in the entrance and kitchen were dirt, cement, wood, or tile. A total of 75 households had paired side-by-side entrance samples collected at either visit. High statistical significance was observed in the side-by-side samples for the two main floor types: dirt and cement. For the two sampling visits combined, the Pearson correlation coefficient for dirt was 0.83 (*p* < 0.0001) and was 0.91 (*p* < 0.0001) for cement, indicating strong agreement of quantitative results. The weighted Kappa statistic was also used to determine agreement when results were evaluated via semi-quantitative analysis, and these findings for both dirt and cement demonstrated substantial to almost perfect agreement. There were too few households with wood floors to power the stratified analysis for this floor type. There were 124 households with paired samples between the entrance and kitchen at either visit. High bacterial loads were identified in homes from human, environmental (flooding), and animal (chicken) contributions as previously published in Exum et al. [7], with statistically significant mean differences of 3.40 log_10_ *E. coli* CFU per 30 by 30 cm sample in the entrance and 3.91 log_10_ *E. coli* CFU (SD = 1.00) (*p*-value = 0.005) in the kitchen. Results for the spatial heterogeneity between the entrance and kitchen floor locations within the same household were significant only when the floor type for those two locations was the same. Comparisons between the entrance and kitchen floors with the same floor type indicated moderate agreement for both the Pearson (0.53, *p* < 0.0001) and the weighted Kappa statistic (0.54, *p* < 0.0001), and stratification of analysis by visit did not change the interpretation.

### 3.3. Field Application to Evaluate Heterogeneity within Households across Time

Table 2 provides the temporal agreement of results for the recovery of *E. coli* from the same household surface location at a three-month interval between the first and second visits. A total of 47 households were sampled during the first visit and then resampled during the second visit. At the entrance, agreement was moderate for both according to the Pearson correlation coefficient (0.50, *p* < 0.001) and the weighted Kappa statistic (0.41, *p* < 0.01). In the kitchen for all floor types, agreement was weak to fair for both the Pearson correlation coefficient (0.37, *p* < 0.001) and the weighted Kappa statistic (0.45, *p* < 0.01). When dirt floors in the kitchen were compared, agreement was also weak to fair for both the Pearson correlation coefficient (0.38, *p* < 0.05) and the weighted Kappa statistic (0.37, *p* < 0.05).

## 4. Discussion

This study demonstrates high efficiency for the recovery of *E. coli* bacteria off of a dry electrostatic cloth in a laboratory setting. For the laboratory experiments, a Nalidixic acid-resistant laboratory strain of *E. coli* was used to reduce the potential of endogenous bacteria impacting the results. The field application of the sampling method demonstrates strong agreement of side-by-side results, moderate agreement for results from different spatial locations within a home at the same time, and weaker agreement for results from floor samples taken at a three-month interval from the same location. Our findings suggest that this method can be utilized in households with high bacterial loads using either continuous (quantitative) or categorical (semi-quantitative) data.

Most prior methods for the collection and transport of *E. coli* from environmental surfaces have used wet swab sampling systems. The dry cloth collection system for *E. coli* developed in this study improves both the recovery and logistical aspects of wet swabs. The dry cloth recovery method achieved near perfect (105% (95% CI: 98%, 113%)) sampling efficiency in the laboratory setting compared to the Moore et al. [5] study, with an observed average sampling efficiency of 50% for *E. coli* from directly inoculated swabs pre-moistened with a variety of swab wetting solutions. This dry approach eliminates the logistical field challenge of maintaining sterile sampling devices that are immersed in a wetting solution prior to sampling. Using the dry cloth approach, the 134 cm^2^ dry cloths can be transported in sterile autoclaved packets with their seal broken just prior to sampling, substantially reducing the potential of cross-contamination. The cost per sample for the wet swab sampling system, which typically consists of a tube, transport medium, and swab with integrated lid, is usually between US $1–2. By contrast, the cost per quarter-sized Swiffer™ dry cloth and 700-mL Whirl-Pak bag for transport is approximately US $0.25. Another strength of the study was the logistical and operational feasibility of the membrane filtration enumeration method in low-income settings where laboratory and waste elimination requirements are often challenging. Other culture-based techniques to analyze bacteria recovered following surface sampling include spread plating, which is only feasible with high titer samples (often not the case with surface sampling) due to the limited volume of sample (<0.5 mL) that can be spread on an agar plate. The use of a most probable number approach such as the commercially available IDEXX Colilert Test, which uses proprietary defined substrate technology (IDEXX, Westbrook, ME, USA), can provide a dynamic range of 1–2000 *E. coli* per sample, but requires a dedicated proprietary sealer and plastic ware which is challenging to dispose of in an environmentally sustainable way. In addition, using membrane filtration and filter plating on media can be less costly and more quantitative than the most probable number provided by the IDEXX system, allowing for additional samples to be tested.

The finding of a slight to moderate weakening of agreement of results over space and time suggests that a multi-site household sampling strategy is necessary to improve environmental exposure assessment for epidemiologic assessment of links with human health outcomes. The heterogeneous distribution of fecal indicator bacteria across environmental reservoirs is well established in both studies of fecal contamination on beach sand [15,16,17] and within households [7,9,18,19,20]. In Exum et al. [7] the kitchen areas of the Peruvian households had statistically significantly higher levels of *E. coli* bacteria than the entrance area; along with the results reported here, this indicates the importance of a sampling strategy to include multiple sites within each household to reduce potential bias due to surface traffic and area use. To further reduce variability in *E. coli* CFU counts, dry cloth samples should be taken at the same time of day over as short a time window as possible so that CFU counts are comparable.

This method is particularly applicable in the developing world context, where access to improved sanitation is lacking and results in elevated levels of fecal contamination. Limitations that temper the generalizability of these findings include that the laboratory experiment and field application were performed in the context of heavy *E. coli* contamination; results may be different in low contamination settings. Additionally, the field application was performed in Iquitos, Peru, where seasonal flooding and the keeping of smallholder livestock (primarily chickens) within households is typical. Recovered *E. coli* isolates were not strain typed, nor was any source attribution (human vs. animal) performed using molecular or epidemiologic methods. It is possible that results will differ in settings less impacted by both human and animal waste inputs.

## 5. Conclusions

The dry cloth method’s high efficacy for the recovery of *E. coli* bacteria in the laboratory setting and the agreement between side-by-side samples measured in the field application justify its standardization as a surface sampling method. The dry cloth method presented in this manuscript has the potential to improve upon the more commonly used swabbing technique that has been shown in the literature to have a low sampling efficiency, thus warranting further research on direct comparisons. As progress is made toward Sustainable Development Goal 6, which calls for ending open defecation and achieving universal access to basic sanitation services by 2030, this standardized surface sampling method will allow for comparisons to be made across communities to assess the effectives of various sanitation interventions to reduce fecal contamination and improve human health.

## Figures and Tables

**Table 1:** Spatial sampling agreement of *E. coli* with the dry cloth method—distribution within household.

	Pearson Correlation Coefficient (95% Confidence Interval)			Kappa Statistic with Kappa Interpretation (Agreement) ^c^					
	First Visit	Second Visit	Combined First and Second Visits	First Visit		Second Visit		Combined First and Second Visits	
Side-by-side entrance samples according to floor type									
Dirt	0.89 (0.42, 0.98) **	0.81 (0.66, 0.90) ****	0.83 (0.70, 0.90) ****	0.90 *	Almost Perfect	0.75 ****	Substantial	0.80 ****	Substantial
	*n* = 7	*n* = 36	*n* = 43	*n* = 7		*n* = 36		*n* = 43	
Cement ^a^	0.996 (0.84, 1.00) **	0.88 (0.75, 0.95) ****	0.91 (0.81, 0.96) ****	0.79	Substantial	0.83 ****	Almost Perfect	0.83 ****	Almost Perfect
	*n* = 4	*n* = 24	*n* = 29	*n* = 4		*n* = 24		*n* = 28	
Wood	--	−0.67 (--, --)	−0.63 (−0.99, 0.84)	--	--	−0.76	--	−0.14	Poor
	*n* = 1	*n* = 3	*n* = 4	*n* = 1		*n* = 3		*n* = 4	
All floor types	0.92 (0.74, 0.98) ****	0.83 (0.73, 0.89) **** †	0.84 (0.76, 0.90) ****	0.83 **	Almost Perfect	0.77 ****	Substantial	0.78 ****	Substantial
	*n* = 12	*n* = 63	*n* = 75	*n* = 12		*n* = 63		*n* = 75	
Entrance and kitchen samples									
Same floor type	0.36 (0.06, 0.59) *	0.65 (0.50, 0.79) ****	0.53 (0.37, 0.66) ****	0.40 **	Fair	0.63 ****	Substantial	0.54 ****	Moderate
	*n* = 43	*n* = 50	*n* = 93	*n* = 43		*n* = 50		*n* = 93	
Different floor type	0.23 (−0.28, 0.64)	0.27 (−0.33, 0.71)	0.29 (−0.08, 0.59)	0.06	Slight	0.02	Slight	0.34	Fair
	*n* = 17	*n* = 13	*n* = 30	*n* = 17		*n* = 13		*n* = 30	
All floor types	0.31 (0.06, 0.52) **	0.60 (0.41, 0.74) ****	0.47 (0.32, 0.60) ****	0.31 **	Fair	0.55 ****	Moderate	0.45 ****	Moderate
	*n* = 61 ^b^	*n* = 63	*n* = 124 ^b^	*n* = 61 ^b^		*n* = 63		*n* = 124 ^b^	

* *p* ≤ 0.05; ** *p* ≤ 0.01; *** *p* ≤ 0.001; **** *p* ≤ 0.0001; † Data previously published [7]; ^a^ One household with tile floors was classified as cement given the similar characteristics; ^b^ One kitchen floor observation was missing data for floor type; ^c^ According to criteria by Landis and Koch [13].

**Table 2:** Temporal sampling agreement of *E. coli* with the dry cloth method—across three months’ time within household.

	Pearson Correlation Coefficient (95% Confidence Interval)	Kappa Statistic with Kappa Interpretation (Agreement) ^c^	
Entrance samples between first and second visits			
Dirt	0.31 (−0.12, 0.64)	0.23	Fair
*n* = 23			
Cement	0.42 (−0.06, 0.74)	0.22	Fair
*n* = 18			
All ^a,b^	0.50 (0.25, 0.69) ***	0.41 **	Moderate
*n* = 47			
Kitchen samples between first and second visits			
Dirt	0.38 (0.03, 0.65) *	0.37 *	Fair
*n* = 30			
Cement	0.62 (−0.39, 0.95)	−0.05	Poor
*n* = 6			
All ^a,b^	0.37 (0.10, 0.60) **	0.45 **	Moderate
*n* = 47			

* *p* ≤ 0.05; ** *p* ≤ 0.01; *** *p* ≤ 0.001; **** *p* ≤ 0.0001; ^a^ Includes heterogeneous floor type comparisons between surface samples; ^b^ Wood floor type sample size too small to show for temporal measurements; ^c^ According to criteria by Landis and Koch [13].
